# Linking the Peptidoglycan Synthesis Protein Complex with Asymmetric Cell Division during *Bacillus subtilis* Sporulation

**DOI:** 10.3390/ijms21124513

**Published:** 2020-06-25

**Authors:** Katarína Muchová, Zuzana Chromiková, Imrich Barák

**Affiliations:** Department of Microbial Genetics, Institute of Molecular Biology, Slovak Academy of Sciences, Dúbravská cesta 21, 845 51 Bratislava, Slovakia; katarina.muchova@savba.sk (K.M.); zuzana.chromikova@savba.sk (Z.C.)

**Keywords:** *Bacillus subtilis*, sporulation, peptidoglycan, SpoIIE, GpsB

## Abstract

Peptidoglycan is generally considered one of the main determinants of cell shape in bacteria. In rod-shaped bacteria, cell elongation requires peptidoglycan synthesis to lengthen the cell wall. In addition, peptidoglycan is synthesized at the division septum during cell division. Sporulation of *Bacillus subtilis* begins with an asymmetric cell division. Formation of the sporulation septum requires almost the same set of proteins as the vegetative septum; however, these two septa are significantly different. In addition to their differences in localization, the sporulation septum is thinner and it contains SpoIIE, a crucial sporulation specific protein. Here we show that peptidoglycan biosynthesis is linked to the cell division machinery during sporulation septum formation. We detected a direct interaction between SpoIIE and GpsB and found that both proteins co-localize during the early stages of asymmetric septum formation. We propose that SpoIIE is part of a multi-protein complex which includes GpsB, other division proteins and peptidoglycan synthesis proteins, and could provide a link between the peptidoglycan synthesis machinery and the complex morphological changes required for forespore formation during *B. subtilis* sporulation.

## 1. Introduction

Spore formation is an extreme response of *Bacillus subtilis* to unfavorable conditions. Sporulation is costly in terms of time and energy, and thus entry into this developmental pathway must be precisely controlled. Sporulation begins with an asymmetric cell division, which produces two unequal daughter cells, a larger mother cell and a smaller forespore. Later, the forespore is engulfed by the mother cell, and the two cells cooperate in the formation of a thick proteinaceous shell, a spore coat. In the final stage, the mature spore is released from the lysing mother cell. The spore can then lie dormant indefinitely and germinate when suitable conditions for growth are restored [1,2].

The first clear morphological event in sporulation is the formation of an asymmetric septum. Elevated levels of FtsZ and SpoIIE play important roles in effecting the switch from mid-cell division to asymmetric division [3,4], and it was recently observed that the division protein DivIVA also takes part in this process and directly interacts with SpoIIE [5]. In the next stage, one polar Z-ring dissolves and the other is transformed into a division septum. SpoIIE is indispensable for efficient asymmetric septation and thus progression of the sporulation process. SpoIIE is a large membrane protein which consists of three main domains: an N-terminal domain (domain I, residues 1–330) is formed by 10 membrane spanning segments; a central domain (domain II, residues 331–589), which is thought to be involved in interaction with FtsZ; and a C-terminal domain (domain III, residues 590–827), which is a PP2C-type phosphatase [6,7]. SpoIIE fulfills several roles in the process of spore formation. First, it is required for asymmetric septum formation and is an integral component of the asymmetric septum [8,9]. Second, by dephosphorylating the anti-σ factor antagonist SpoIIAA, SpoIIE activates the forespore-specific transcription factor σ^F^. Despite numerous studies, the mechanism by which σ^F^ is specifically activated only in the forespore is still not fully understood [10,11,12,13]. A possible third role for SpoIIE is connected with forespore engulfment and arises from the observation that it is recaptured to the forespore face of the polar septum where it interacts with SpoIIQ. SpoIIQ anchors SpoIIE to the engulfing membrane where it may participate in peptidoglycan remodeling [14]. The idea that it might be involved in peptidoglycan remodeling seems to be reinforced by the recent observation that SpoIIE interacts with the morphogenic protein RodZ, a component of the peptidoglycan synthesizing machinery [15]. Although formation of the asymmetric septum requires the same set of division proteins as the vegetative septum, the asymmetric septum is significantly different from it. First, it contains SpoIIE as an integral component; second, it is much thinner than the vegetative septum [8,9]. Deletion of *spoIIE* causes defective sporulation and gives rise to aberrantly thick asymmetric septa similar to vegetative septa [8], which appear at different positions than in wild-type cells [16]. Cells without SpoIIE cannot form spores. How and even whether SpoIIE is directly responsible for the thinner sporulation septa is not clear. 

The peptidoglycan comprising a major component of the bacterial cell wall is synthesized during cell growth and division. The coordinated action of two large protein complexes, the elongasome and the divisome, is responsible for peptidoglycan synthesis. The first directs insertion of peptidoglycan along the long axis of the cell while the second acts at the site of division. The synthesis of septal peptidoglycan is under the control of the tubulin-like protein FtsZ. At the beginning of cell division, FtsZ is polymerized into a structure called the Z-ring. The Z-ring then recruits over 20 other division proteins to form a divisome. Among these are proteins required for peptidoglycan synthesis, such as penicillin binding proteins (PBPs), and various regulatory proteins, including DivIVA, MinC, MinD and GpsB. GpsB was first described in *B. subtilis* as a paralog of division protein DivIVA. GpsB is widely conserved in the *Firmicutes* phylum and in low G+C Gram-positive bacteria and has moderately different functions in different species [17,18,19,20]. In *B. subtilis,* GpsB functions to shuttle PBP1, the major transglycosylase/transpeptidase, away from the cell pole to the sidewall for elongation, while EzrA, an FtsZ regulator, serves to return PBP1/GpsB back to the septum for division [17]. Generally, it is thought that GpsB is an adaptor for the peptidoglycan synthesizing enzymes, PBPs, and directs them to the protein complexes responsible for cell wall synthesis during cell elongation and cell division [21]. Deletion of *gpsB* has no effect on cell division; however, it does have a synthetic effect when combined with mutations in the cell division genes *ftsA* or *ezrA* [17,20]: when *ezrA* and *gpsB* are deleted, cells become elongated and prone to lysis [17]. GpsB is also not essential for sporulation, as either polar septum formation or sporulation efficiency was affected in *gpsB* mutants [20]. 

In this study, we confirm that GpsB localizes to the polar septum during sporulation and we show that it co-localizes with SpoIIE during the early stages of asymmetric septum formation. We demonstrate that GpsB interacts directly with SpoIIE. We hypothesize that GpsB continues to fulfill its growth role as an important cell cycle regulator and adaptor for cell wall enzymes during asymmetric cell division. In addition, we suggest that, since GpsB is not essential for sporulation, it does this during the early stages of sporulation through direct contact with the indispensable sporulation protein SpoIIE. Finally, we propose that a multi-protein complex, including SpoIIE, division proteins and proteins involved in peptidoglycan synthesis, controls asymmetric septum formation. 

## 2. Results and Discussion

### 2.1. GpsB Interacts with SpoIIE

The bacterial cell wall is responsible for cell integrity and the maintenance of cell shape. Peptidoglycan, a three-dimensional network of glycan strands cross-linked by peptide bridges, comprises a major component of the cell wall [22]. Peptidoglycan is synthesized during cell elongation when new peptidoglycan is inserted in the lateral walls, and during division, when it is synthesized at the division septum. PBPs accomplish the synthesis of peptidoglycan. It was recently shown that GpsB, a cytoplasmic protein, is a main regulator of peptidoglycan biosynthesis in low G+C Gram-positive bacteria; GpsB may function as an adaptor that connects PBPs to various multiprotein complexes such as the elongasome or the divisome [21]. 

**Figure 1:**
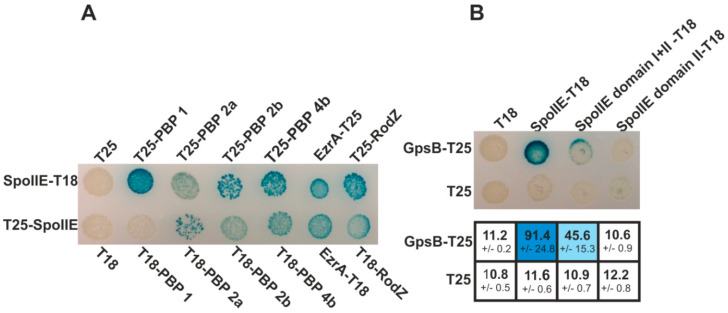
Bacterial two-hybrid analysis of SpoIIE. (**A**) Interactions of SpoIIE with proteins involved in peptidoglycan biosynthesis. *E. coli* strain BTH101 (*Δcya*) was co-transformed with plasmids encoding the indicated fusions to adenylate cyclase fragments T18 and T25. Colonies were spotted onto selective plates containing IPTG and X-Gal. A blue color indicates a positive interaction between each pair of fusion proteins; (**B**) Bacterial two-hybrid interactions of GpsB with SpoIIE and SpoIIE domains. After plasmids co-transformation, colonies were spotted onto selective plates containing IPTG and X-Gal. A blue color indicates a positive interaction between each pair of fusion proteins. The interactions were quantified using a β-galactosidase assay. Numbers show Miller units of activity and represent the mean ± standard deviation of at least three independent measurements. The interaction strength is indicated by the intensity of the blue color. The β-galactosidase activity values in the table correspond to the interactions shown in the upper panel.

In this work, we sought to determine how peptidoglycan synthesis is linked to asymmetric cell division during *B. subtilis* sporulation. We focused on the early stages of sporulation, when such cell division takes place. It is known that the same set of proteins is involved in both the formation of the vegetative division septum and the asymmetric sporulation septum (see recent review [23]). In addition, a sporulation specific protein, SpoIIE, is known to be indispensable for successful sporulation. The sporulation septum in wild-type cells, besides its positioning, also differs from the vegetative septum in its peptidoglycan content: it is thinner than the vegetative one. Since SpoIIE is probably responsible directly or indirectly for the structure of the sporulation septum, we searched for interactions between SpoIIE and some proteins involved in peptidoglycan biosynthesis. Using a bacterial two-hybrid (BACTH) system, we found significant interactions between SpoIIE and proteins involved in peptidoglycan biosynthesis (Figure 1A) in addition to the known interaction between SpoIIE and RodZ [15]. SpoIIE’s observed interactions with EzrA, RodZ and all tested PBPs indicate that SpoIIE is involved in the process of peptidoglycan biosynthesis. The BACTH system does have its limitations; however, when used for screening interactions between membrane proteins, the possibility that the tested proteins are simply accumulating in the membrane of *Escherichia coli*, bringing the T25 and T18 domains of adenylate cyclase into close enough proximity for synthesis of cyclic AMP and thereby producing spurious positive interactions, cannot be excluded. On the other hand, it was previously shown that EzrA and RodZ localize to the asymmetric septum [15,24]. Moreover, PBP1 and PBP2b are also transiently localized to this septum [25,26]. Even though the localization of PBP4b during sporulation has not yet been determined [27], it is known that PBP4b is a sporulation specific class B PBP [28]. Taken together, these data suggest that all these proteins are probably components of the sporulation division and peptidoglycan synthesizing machinery and may be in direct contact with SpoIIE. Despite these indications, additional biochemical methods, similar to those used for the RodZ–SpoIIE interaction [15], are needed to confirm these direct interactions. Recently, GpsB, a cytosolic protein, was shown to function as a linker between the major PBPs and protein complexes involved in various cell processes, including cell division and elongation [21]. We, therefore, asked if GpsB might also fulfill this role during sporulation and target PBPs to the asymmetric septum. We initially employed a BACTH system to test for an interaction between GpsB and SpoIIE, a major component of the sporulation septum. We found a moderate interaction between GpsB and SpoIIE (Figure 1B). To analyze the contact of SpoIIE and GpsB in more detail, we also tested for interactions between individual SpoIIE domains (cloned in fusion with both domains of adenylate cyclase [15]) and GpsB. Unfortunately, when SpoIIE domain III was cloned separately, we observed significant self-activation when combining these plasmids with the relevant empty vectors, which makes these constructs unsuitable for further interaction screening. We detected only weak interactions between GpsB and SpoIIE domains I+II (Figure 1B).

**Figure 2:**
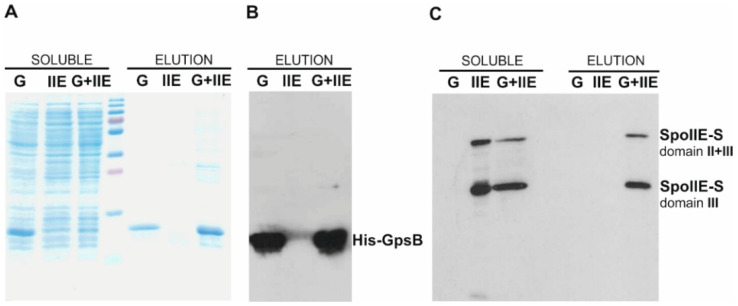
Pull-down assay of proteins isolated from *Escherichia coli* BL21 (DE3). GpsB was His-tagged while cyt-SpoIIE (SpoIIE domain II+III) was S-tagged. The pull-down assay was performed on a Ni Sepharose HP column. (**A**) SDS-PAGE gel stained with Coomassie Brilliant Blue. Lanes soluble G, IIE and G+IIE show cell lysates containing soluble His-GpsB, cyt-SpoIIE-S and His-GpsB+cyt-SpoIIE-S. Lane marked elution G shows His-GpsB eluted with 1 M imidazole. Lane marked elution IIE shows cyt-SpoIIE-S eluted with 1 M imidazole. Lane marked elution G+IIE shows His-GpsB +cyt-SpoIIE-S eluted with 1 M imidazole; (**B**) Western blot of eluted fractions. Eluted proteins were probed with an anti-His-tag monoclonal antibody. The lanes correspond to the lanes on the right-hand side of panel A; (**C**) Western blot of eluted fractions. Eluted proteins were probed with an anti-S-tag monoclonal antibody. Lane soluble G shows that no GpsB is detected in a cell lysate containing soluble His-GpsB. Lanes soluble IIE and G+IIE show soluble cyt-SpoIIE-S (SpoIIE-S domain II+III) in cell lysates of cyt-SpoIIE-S and His-GpsB+cyt-SpoIIE-S. Lanes marked elution G and IIE show that no cyt-SpoIIE-S is present in the eluted fractions of His-GpsB or cyt-SpoIIE-S when produced alone. cyt-SpoIIE-S (SpoIIE-S domain II+III) is detected only in those fractions where it is pulled down with His-GpsB (lane elution G+IIE).

To confirm the interaction between SpoIIE and GpsB, we performed a pull-down assay using proteins expressed and purified from *E. coli*. We used a previously described soluble S-tagged fragment of SpoIIE cyt-SpoIIE-S (SpoIIE-S domain II+III) comprising residues 332–827 representing the complete cytosolic part of SpoIIE [15] and a His-tagged GpsB prepared in this work (see Materials and Methods). His-GpsB can be affinity purified on a Ni^2+^ column; an interacting cyt-SpoIIE-S can then be pulled down and subsequently detected using a fused S-tag. SDS-PAGE revealed that both proteins were expressed in *E. coli* and were soluble (Figure 2A,C soluble, lanes G, IIE, G+IIE). The proteins were then purified on a Ni^2+^ column (Figure 2A–C elution lanes G, IIE, G+IIE). As can be seen in Figure 2C, cyt-SpoIIE-S (SpoIIE-S domain II+III) is not detected in the elution fraction when produced alone (Figure 2C, elution lane IIE), but is pulled down with His-GpsB (elution lane G+IIE). This result suggests that the cytosolic protein GpsB directly associates with the cytosolic part of SpoIIE. 

Recently, a crystal structure of GpsB bound to peptides from the cytoplasmic regions of PBP1 was solved [21]. This structure revealed the conserved motif in PBP1 orthologues that is required for GpsB–PBP1 complex formation [21]. A similar motif was found in the sporulation specific proteins of unknown function, YpbE and YrrS, which are possible members of the GpsB interactome [21]. GpsB also binds to the serine/threonine protein kinase PrkC and is phosphorylated by this kinase at a single site, Thr-75 [29]. In addition, EzrA and MreC were found to be GpsB protein partners [17], although their key interaction residues are currently not known [30]. The observed interaction of GpsB with SpoIIE extends its interactome and confirms its role as a mediator in the formation of multi-protein complexes [30]. Interestingly, all known GpsB interaction partners are membrane-associated or transmembrane proteins [17,21,29]. This suggests that GpsB has a specific role as a protein linker. 

### 2.2. GpsB Co-Localizes with SpoIIE during Sporulation 

**Figure 3:**
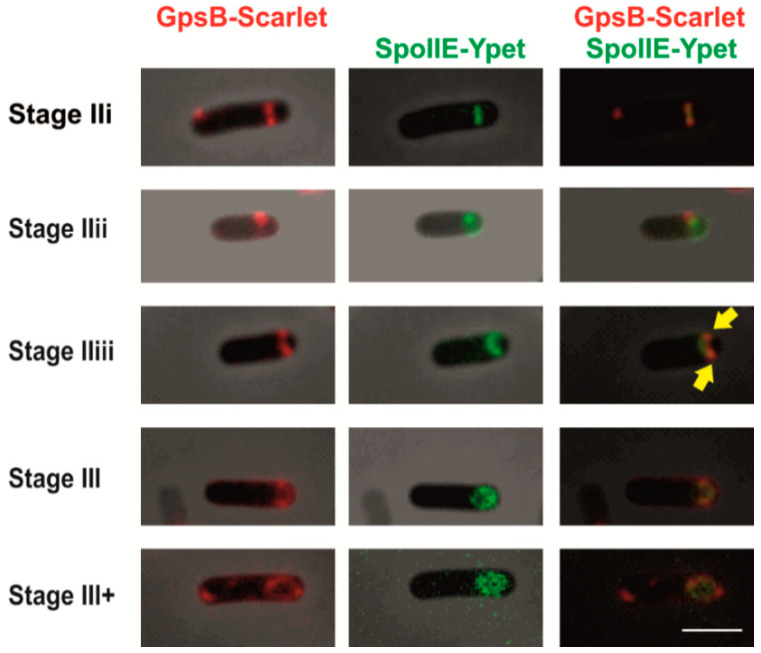
Localization of SpoIIE and GpsB in sporulating cells. Differential localization of SpoIIE-Ypet and GpsB-mScarlet (KM1324) in cells during the early stages of sporulation. Cells were harvested 2 h after the onset of stationary phase. Column GpsB-Scarlet: phase contrast + mScarlet fluorescence, column SpoIIE-Ypet: phase contrast + SpoIIE-Ypet fluorescence (SpoIIE-Ypet signal has been false-colored green), column GpsB-Scarlet + SpoIIE-Ypet: phase contrast + GpsB-mScarlet fluorescence +SpoIIE-Ypet fluorescence (SpoIIE-Ypet signal has been false-colored green). Yellow arrows show the accumulation of GpsB-mScarlet at the leading edges of the forespore engulfing membrane. The scale bar represents 2 μm.

Successful sporulation as an adaptive response to non-favorable environmental conditions requires the cooperative action of hundreds of different proteins. GpsB, as shown previously, is not essential for sporulation, and *ΔgpsB* cells sporulate with similar efficiency as wild-type cells [20]. The first localization experiments revealed that GFP-GpsB produced from a xylose inducible promoter localizes as a single band close to one of the cell poles in early sporulating cells [20]. In addition, in some cells a second band near the other cell pole could also be seen [20]. To follow GpsB localization in more detail, we prepared several strains in which GpsB is fused either to Ypet, a photostable derivative of YFP [31] (KM1202), or to mNeongreen (KM1309) or mScarlet (KM1322) and is produced under the control of its native promoter. In general, we observed a similar GpsB pattern of localization (Figure 3) as described previously [20]. A detailed analysis revealed that GpsB-mScarlet accumulates at the straight, and subsequently the slightly curved, polar septum (Figure 3 stage IIi and IIii). Later, two highly concentrated foci of GpsB-mScarlet at both leading edges of the forespore engulfing membrane were observed (Figure 3 stage IIiii). As sporulation proceeds, GpsB-mScarlet localizes along the engulfing membrane and around the forespore (Figure 3 III and III+). To compare GpsB localization with the localization of SpoIIE, the main asymmetric septum constituent, we prepared a strain expressing *gpsB*-*mscarlet* under the control of its native promoter and SpoIIE fused to Ypet produced from its native promoter (KM1324) (Figure 3). GpsB-mScarlet and SpoIIE-Ypet display a similar pattern of localization (Figure 3). As sporulation begins, GpsB-mScarlet localizes at polar division sites together with SpoIIE-Ypet (Figure 3 stage IIi and IIii). A detailed comparison of the septal localization of GpsB and SpoIIE revealed that their localization differs at later stages of development. Specifically, at stage IIiii, GpsB accumulates in foci at both leading edges of the forespore engulfing membrane (Figure 3). On the other hand, in this stage, SpoIIE is recaptured by SpoIIQ on the forespore face of the polar septum. Finally, as engulfment is completed, and cells reach stage III of sporulation, the GpsB-mScarlet signal around the forespore (GpsB also seems to be localized to the outside face of the forespore) and the SpoIIE-Ypet signal around and inside the forespore can be observed (Figure 3 III and III+). Taken together, this comparison of the GpsB and SpoIIE localizations reveals that these two proteins co-localize during the early stages of asymmetric septum formation. This is consistent with the idea that GpsB and SpoIIE are present in the same functional complex, and together with the divisome proteins, directly participate in the establishment of the asymmetric septum. Their co-localization is thus most apparent during the early stages (Figure 3 stage IIi) of asymmetric septum formation. Later, when SpoIIE is released and subsequently recaptured to the polar septum (Figure 3 stage IIii and IIiii), GpsB seems to accumulate in foci at both ends of the septum (Figure 3 stage IIiii, yellow arrows). Localization of GpsB at these leading edges of the engulfing membrane may be determined by its function during engulfment when it probably participates in peptidoglycan synthesis and remodeling by recruiting PBPs. 

### 2.3. Localization of SpoIIE and GpsB Is Mutually Independent

**Figure 4:**
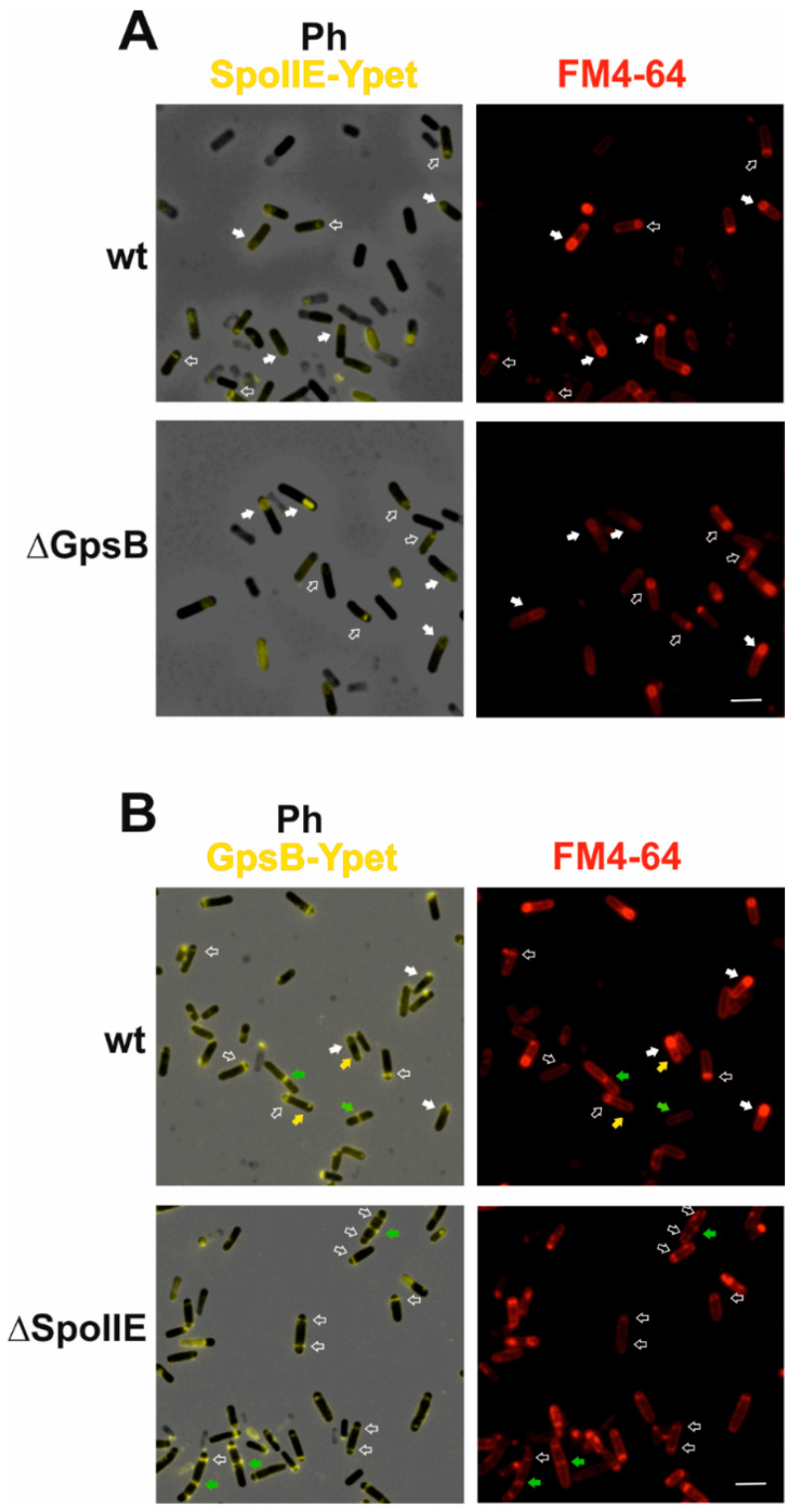
Localization of SpoIIE in a *ΔgpsB* strain and GpsB in *ΔspoIIE*. Cells were harvested 2 h after the onset of stationary phase. Membranes were stained with FM4-64 (red). (**A**) The panel marked Ph SpoIIE-Ypet shows phase contrast merged with SpoIIE-Ypet fluorescence; the panel FM4-64 shows membranes visualized using FM4-64. Empty arrows show SpoIIE-Ypet localization in stage II; full arrows, SpoIIE-Ypet localization in stage III and later stages; (**B**) The panel marked Ph GpsB-Ypet shows phase contrast merged with GpsB-Ypet fluorescence; the panel FM4-64 shows membranes visualized using FM4-64. Empty arrows show GpsB-Ypet localization in stage II; full arrows show GpsB-Ypet localization in stage III and later stages. Yellow arrows indicate the localization of GpsB-Ypet at the second polar position. Green arrows show the localization of GpsB-Ypet in vegetative septa. The scale bars represent 2 μm.

To test whether SpoIIE localization is influenced by the absence of GpsB, we prepared a *gspB* deletion strain in which SpoIIE-Ypet is expressed under the control of its own promoter (KM1327). In this background, SpoIIE-Ypet localizes similarly as in wild-type cells (Figure 4A). Its fluorescence signal was clearly visible at polar septa (Figure 4A); in some cells proceeding into stage III, the signal was around the forespore (Figure 4A). Taken together, SpoIIE localization is not disturbed in those cells lacking GpsB. Since the determinants of GpsB localization to septal sites are not known [30], we were curious whether SpoIIE, which is required for the high efficiency initiation of asymmetric septum formation [8,32], might not direct the localization of GpsB to the asymmetric septum. We prepared a *spoIIE* deletion strain expressing *gpsB-ypet* from its own promoter (KM1325). Under sporulation conditions, we observed the GpsB-Ypet fluorescence signal at polar septa (Figure 4B). We observed more cells with GpsB-Ypet localized at both polar septa in a *ΔspoIIE* background (Figure 4B) than in the wild-type background; an increase in cells with two polar septa is consistent with the previously described disporic phenotype of the *ΔspoIIE* mutant [32]. While in the wild-type background, some cells proceeded into stage III and later stages (Figure 4B); sporulation in *ΔspoIIE* terminated at stage II as expected. Altogether, it seems that the localizations of SpoIIE and GpsB to the asymmetric sporulation septum are mutually independent. It is known that the localization of SpoIIE to polar division sites depends on the earliest components of the divisome, division proteins FtsZ and FtsA [4,33]. During vegetative growth, GpsB has been found to switch between the sidewalls and division septum [17,34]. Although GpsB is considered to be a late division protein, the GpsB localization determinants are unknown [30]. At the beginning of sporulation, GpsB seems to follow the changing position of the divisome, and localizes to the polar division sites [20], as was also observed in this work.

### 2.4. SpoIIE and GpsB Are Components of a Multi-Protein Complex

**Figure 5:**
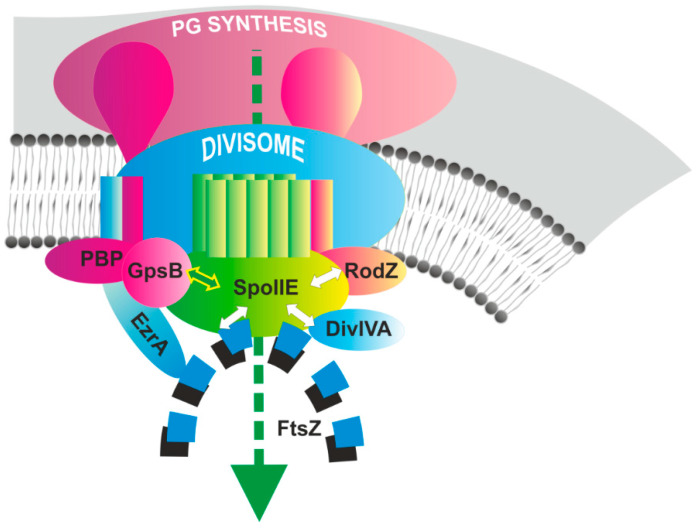
The multi-protein complex required for sporulation septum formation. The model shows the crucial role of SpoIIE (green) in the process of septum formation and its interaction partners. The dotted line across the image ending with an arrow represents the plane of upcoming septation. The divisome is colored blue and includes all division proteins (FtsA, SepF, DivIB, DivIC, FtsL, FtsW); the FtsZ ring is depicted by small blue squares; the division proteins DivIVA and EzrA are also colored blue. The peptidoglycan synthesis machinery is colored pink; GpsB, RodZ and the PBPs that are part of the peptidoglycan synthesis machinery are also colored pink. The proteins which have been shown to directly interact with SpoIIE (FtsZ, DivIVA, RodZ, GpsB) are connected to SpoIIE with double-headed arrows. A green arrow is used to indicate the newly-discovered interaction between SpoIIE and GpsB.

Taking into account our current results and those from previous studies (see recent review [23]), we propose that a multi-protein complex is required for efficient asymmetric septum formation and successful sporulation with an essential role belonging to SpoIIE (Figure 5). 

As was demonstrated previously, at the onset of sporulation SpoIIE is targeted to polar division sites in an FtsZ-dependent manner, and co-localizes there with the polar Z-rings, probably through a direct interaction [4,35]. Recently, it was demonstrated that cyt-SpoIIE and FtsZ co-polymerize *in vitro* and form very stable polymers [36]. Another essential component of this complex is DivIVA, which, in addition to its role in chromosome segregation at the onset of sporulation [37,38], interacts with SpoIIE and is required for its proper functioning [5]. *divIVA* null mutants are defective in asymmetric septation, and σ^F^ is also prematurely activated in a compartment-nonspecific manner [5]. Sporulation efficiency in *divIVA* null mutants is markedly reduced about 5% compared to the wild type [39] (Table 1). 

In addition to these proteins, other division proteins, which participate in vegetative cell division, and peptidoglycan synthesis proteins, are also components of this complex (Figure 5). Recently, we showed that the morphogenic protein RodZ may be part of this complex and is in direct contact with SpoIIE [15]. In this work, we showed that the GpsB adaptor protein [21] is another SpoIIE protein partner. Whether EzrA and the PBPs, whose interactions with SpoIIE were detected using a two-hybrid system, are additional SpoIIE partners remains to be verified. We propose that the assembly of this multi-protein complex, the asymmetric divisome together with the peptidoglycan biosynthesis machinery, is regulated on various levels. However, the necessity of individual proteins in this complex differs, with some being indispensable for asymmetric septum formation and others producing no detectable effect on the sporulation process when they are absent (Table 1). When EzrA is missing, the sporulation efficiency is not significantly changed and cells sporulate at the wild-type level [24]. Similarly, *gpsB* null mutant cells sporulate efficiently, so GpsB is also not necessary for spore development [20]. PBP2b is the only PBP that is essential for both vegetative growth and sporulation in *B. subtilis* [26]. A *ponA*-null mutant (PBP1) has been reported to have a markedly reduced frequency of asymmetric septum formation, resulting in reduced sporulation efficiency, only 14% of the wild type [25]. Finally, as we showed previously, in *rodZ* mutant cells sporulation efficiency is significantly reduced to as low as 0.3–0.9% in minimal medium or 24–30% in DSM medium [15]. In addition, RodZ is involved in stabilizing SpoIIE in the septum. 

## 3. Materials and Methods

### 3.1. Media and General Methods

*Escherichia coli* strains were grown in LB media [41]; *B. subtilis* cells were grown in Difco sporulation medium (DSM) [42]. When required, media were supplemented with chloramphenicol (5 μg·mL^−1^), kanamycin (10 μg·mL^−1^) or erythromycin (1 μg·mL^−1^) and lincomycin (25 μg·mL^−1^). In general, all molecular biology experiments in *B. subtilis* were done using standard protocols [42].

### 3.2. Bacterial Strains and Plasmids

The bacterial strains used in this study are shown in Table S1; all prepared *B. subtilis* strains are derivatives of *B. subtilis* PY79 [43]; *E. coli* strains MM294 [44] and DH5α (Invitrogen, Waltham, MA, USA) were used for cloning and plasmid isolation. Plasmids used in this study are listed in Table S2; the sequences of the oligonucleotides used in this work are given in Table S3.

To replace *gpsB* at the native locus with *gpsB-ypet*, a PCR-fragment-containing part of *gpsB* (31–98 aa) was digested by KpnI and cloned into the KpnI site of pSGIIE-Ypet [15] exchanging the *spoIIE* part of the construct. pSGgpsB-mneongreen was constructed in two steps. First, *mneongreen* was PCR amplified from expression vector pET14(b+)mneongreen (kind gift from Mark Leake) using the primers mneongreenSKpn and mneongreenEPst and ligated into a pSG1151 vector [45], resulting in pSGmneongreen. The *gpsB* fragment obtained by cutting pSGgpsB-ypet with KpnI was subsequently cloned into pSGmneongreen to create the integration plasmid pSGgpsB-mneongreen. 

To follow the possible co-localization of GpsB and SpoIIE in *B. subtilis,* pUCkangpsB-mscarlet was created. First, a kanamycin resistance gene obtained by the PCR amplification of *kan* from pUK19 [46] (primers kanSH and kanEH) was introduced into pUC19 [47]. Then a PCR fragment of *mscarlet* was amplified from expression vector pET14(b+)mscarlet (kind gift from Mark Leake) using the primers mscarletSKpn and mscarletEPst. After digestion with KpnI and PstI, this fragment was cloned into a similarly cut pUCkan vector. Finally, a *gpsB* fragment obtained by cutting pSGgpsB-ypet with KpnI was cloned into this vector to create the integration plasmid pUCkangpsB-mscarlet.

To analyze the interactions between GpsB and the cytosolic part of SpoIIE using a pull-down method, we used the recombinant plasmid pETspoIIE-S prepared for earlier studies [15] and created pETgpsB. pETgpsB, which contains a His-tagged *gpsB,* was prepared by cloning a *gpsB* PCR fragment (primers gpsBSNd and gpsBEBam) into a pET15b(+) vector (EMD Biosciences, Inc., Novagen, Germany).

### 3.3. Bacterial Two-Hybrid System

The target genes were amplified by PCR from *B. subtilis* PY79 chromosomal DNA. PCR fragments of *gpsB*, *ezrA* and *ponA* were cloned into similarly digested vectors of a BACTH bacterial two-hybrid system [48] to generate plasmids encoding the corresponding proteins fused to the T25 and T18 fragments of adenylate cyclase.

To test for protein–protein interactions, each pair of plasmids was co-transformed into *E. coli* BTH101. Co-transformation mixtures were spotted onto LB plates supplemented with 40 μg·mL^−1^ X-Gal (5-bromo-4-chloro-3-indolyl-β-d-galactopyranoside), 0.5 mM IPTG, 100 μg·mL^−1^ ampicillin and 30 μg·ml^−1^ kanamycin, and grown for 24–48 h at 30 °C. β-galactosidase activity was measured as described by Miller [49] with an extra wash step.

### 3.4. Protein Isolation and Purification

*E. coli* BL21 (DE3) strains harboring expression plasmids were grown in LB medium at 37 °C. When the OD_600_ of the culture reached 0.5, expression of recombinant proteins was induced by the addition of 1 mM IPTG. After 3 h further growth at 37 °C for GpsB or overnight growth at 16 °C for the cytosolic part of SpoIIE, the cells were harvested by centrifugation. Cell pellets were resuspended in lysis buffer (20 mM Tris-HCl, pH 8.0, 150 mM NaCl) before being disrupted by sonication. The lysate was centrifuged at 30,000 rpm for 30 min to remove cell debris. His-tagged proteins were purified using a 1 mL Ni Sepharose HP column (Amersham Biosciences, Little Chalfont, UK). Proteins were eluted with 1 M imidazole. Co-eluted proteins were identified by Coomassie Brilliant Blue staining and by Western blot analysis using monoclonal antibodies against the S-tag and His-tag (EMD Biosciences, Inc., Novagen, Germany).

### 3.5. Fluorescence Microscopy and Image Acquisition

*B. subtilis* cultures were grown as liquid cultures in DSM medium, as described above, and cells were harvested 2 h after the onset of stationary phase. For membrane visualization, the fluorescent dye FM 4–64 (Molecular Probes, Eugene, OR, USA) was used at concentrations of 0.2–1 μg·ml^−1^. Cells were examined under the microscope on 1% agarose covered slides. When it was necessary to increase the cell density, cells were concentrated by centrifugation (3 min at 2500 rpm) and resuspended in a small volume of supernatant prior to examination by microscopy. All images were obtained with an Olympus BX63 microscope equipped with an sCMOS Zyla-4.2P camera (Andor, Oxford Instruments, Belfast, UK). Olympus CellP imaging software and ImageJ software were used for image acquisition and analysis.

## 4. Conclusions

Our work shows a direct contact between the SpoIIE and GpsB proteins during asymmetric cell division in *B. subtilis*. Importantly, SpoIIE also interacts with FtsZ and some cell division proteins such as DivIVA and, likely, EzrA, and with RodZ and PBPs—other proteins involved in peptidoglycan synthesis. We propose that SpoIIE is a crucial link between the asymmetric division protein complex and the peptidoglycan biosynthesis machinery. However, future studies are required to fully understand the roles of other proteins in this complex.

## Figures and Tables

**Table 1:** Sporulation efficiency of selected deletion strains. Sporulation efficiency in percent compared to the wild type; rodZ* represents *rodZ* depletion strain.

Mutation	Sporulation Efficiency (%)	Reference
*spoIIE*	<0.00001	[40]
*divIVA*	5	[39]
rodZ*	24–30	[15]
*ponA* (PBP1)	14	[25]
*gpsB*	100	[20]
*ezrA*	100	[24]

## Supplementary Materials

Supplementary materials can be found at https://www.mdpi.com/1422-0067/21/12/4513/s1, Table S1. Bacterial strains; Table S2. Plasmids; Table S3. Oligonucleotides used in this work.
