# Supplementary material for: Linking the Peptidoglycan Synthesis Protein Complex with Asymmetric Cell Division during Bacillus subtilis Sporulation
Source: Int J Mol Sci. 2020 Jun 25;21(12):4513. doi: 10.3390/ijms21124513 (PMC7349982; doi:10.3390/ijms21124513)
Supplement: Supplementary file 1 [file ijms-21-04513-s001.pdf]

Supplementary Materials

Linking peptidoglycan synthesis protein complex with asymmetric cell division during *Bacillus subtilis* sporulation

K. Muchová, Z. Chromiková and I. Barák*

*Author for correspondence: imrich.barak@savba.sk ; Tel.:+421259307418

Table S1. Bacterial strains

**Strain**	**Genotype**	**Source/reference**
B. subtilis		
PY79	Prototrophic derivative of *B. subtilis* 168	[43]
PY180	*spoIIE*::Tn*917ΩHU7*	[8]
JR46	*gpsB::kan*	[20]
KM1202	p_{gpsB} *gpsB-ypet cat*	this work
KM1309	p_{gpsB} *gpsB-mneongreen cat*	this work
KM1322	p_{gpsB} *gpsB-mscarlet kan*	this work
KM1324	p_{spoIIE} *spoIIE-ypet cat* p_{gpsB} *gpsB-mscarlet kan*	this work
KM1325	p_{gpsB} *gpsB-ypet cat spoIIE::Tn917ΩHU7*	this work
KM1327	p_{spoIIE} *spoIIE-ypet cat gspB::kan*	this work
IB1537	p_{spoIIE} *spoIIE-ypet cat*	[15]
E. coli		
MM294	F^{-} *endA-1 hsdR-1, (rk*$^{-}$*, mk) supE44 thi-1 recA1*	[44]
DH5α	F^{-} *Φ80lacZΔM15 Δ(lacZYA-argF) U169 recA1 endA1 hsdR17 (rK*$^{-}$*, mK*$^{+}$*) phoA supE44 λ– thi-1 gyrA96 relA1*	Invitrogen
BL21(DE3)	*hsdS gal (λcts857 indt Sam7 nin5 lacUV5-T7gene*	Novagen

BTH101	*F^-cya-99 araD139 galE15 galK16 rpsL1(Str^r)hsdR2 mcrA1 mcrB1*	[48]

Table S2. Plasmids

Plasmid	Description	Reference
pSG1151	*bla cat gfpmut1*	[45]
pUK19	*bla kan*	[46]
pET15 b(+)	expression vector used for protein expression, *bla lacI T7 promoter*	Novagen
pUC19	vector with a multiple cloning site (MCS) *bla*	[47]
pSGIIE-Ypet	*bla cat spoIIE (724-827aa)-ypet*	[15]
pSGgpsB-ypet	*bla cat gpsB (31-98aa)-ypet*	this work
pSGgpsB-mneongreen	*bla cat gpsB (31-98aa)-mneongreen*	this work
pUCkangpsB-mscarlet	*bla kan gpsB (31-98aa)-mscarlet*	this work
pETgpsB	*bla lacI pT7 his-gpsB*	this work
pKT25	enable fusion to C-terminal end of adenylate cyclase T25 fragment	[48]
pKNT25	enable fusion to N-terminal end of adenylate cyclase T25 fragment	[48]
pUT18	enable fusion to N-terminal end of adenylate cyclase T18 fragment	[48]
pUTC18	enable fusion to C-terminal end of adenylate cyclase T18 fragment	[48]
pKTgpsB	*p_{lac}-T25-gpsB-kan*	this work
pKNTgpsB	*p_{lac}-gpsB-T25 kan*	this work

pUTgpsB	p_{lac}*-gpsB-T18 bla*	this work
pUTCgpsB	p_{lac}*-T18-gpsB bla*	this work
pKTIIE	p_{lac}*-T25-spoIIE kan*	[15]
pKNTIIE	p_{lac}*-spoIIE-T25 kan*	[15]
pUTIIE	p_{lac}*-spoIIE-T18 bla*	[15]
pUTCIIE	p_{lac}*-T18-spoIIE bla*	[15]
pKTnctIIE	p_{lac}*-T25- spoIIE domain I,II kan*	[15]
pKNTnctIIE	p_{lac}*-spoIIE domain I,II-T25 kan*	[15]
pUTCnctIIE	p_{lac}*-T18-spoIIE domain I,II bla*	[15]
pUTnctIIE	p_{lac}*-spoIIE domain I,II T18 bla*	[15]
pKTctIIE	p_{lac}*-T25- spoIIE domain II kan*	[15]
pKNTctIIE	p_{lac}*-spoIIE domain II-T25 kan*	[15]
pUTCctIIE	p_{lac}*-T18-spoIIE domain II bla*	[15]
pUTctIIE	p_{lac}*-T18-spoIIE domain II bla*	[15]
pKNTezrA	p_{lac}*-ezrA-T25 kan*	this work
pUTezrA	p_{lac}*-ezrA-T18 bla*	this work
pKTponA	p_{lac}*-T25-ponA kan*	this work
pUTCponA	p_{lac}*-T18-ponA bla*	this work

Table S3. Oligonucleotides used in this work

**Primer**	**Sequence 5′- 3′**
gpsBSKpnI	GCTGAATATGGTACCGACAAATTTTTAGATATGATTATTAAGGATTATG
gpsBEKpnI	GATGATGATGGTACCCCAATCATAAAGCTTGCTGCCAAAAAC
gpsBBamF4	GTAGTAGTAGGATCCCATGCTTGCTGATAAAGTAAAGCTTTCTG
gpsBEcoR	GTAGTAGTAGAATTCGAATCATAAAGCTTGCTGCCAAAAAC
gpsBKTEcoR	GTAGTAGTAGAATTCTCAATCATAAAGCTTGCTGCCAAAAAC
ezrABamF2	GATGATGATGGATCCCATGGAGTTTGTCATTGGATTATTAATTG

ezrAEcoR	GATGATGATGAATTCGAAGCGGATATGTCAGCTTTGATTTTTTC
ezrAKTEcoR	GATGATGATGAATTCCTAAGCGGATATGTCAGCTTTGATTTTTTC
ponABamF	CTACTACTAGGATCCCATGTCAGATCAATTTAACAGCCGTG
ponAEcoR2	CTACTACTAGAATTCGAATTTGTTTTTTCAATGGATGATGAG
ponAKTEcoR	CTACTACTAGAATTCTTAATTTGTTTTTTCAATGGATGATGAG
gpsBSNd	CATGCGTCCCATATGCTTGCTGATAAAGTAAAGC
gpsBEBam	GCACCGGATGGATCCTCAATCATAAAGCTTGCTGCCAAAAAC
mscarletSKpn	GCGAACGCAGGTACCATGGTCTCCAAAGGAGAG
mscarletEPst	GCGAACGCACTGCAGTTATTTATACAGCTCATCCATAC
mneongreenSKpn	GCGAACGCAGGTACCATGGTGAGCAAGGGCGA
mneongreenEPst	GCGAACGCACTGCAGCTACTTGTACAGCTCGTC
kanSHind	GATGATGATAAGCTTTCGCCGTATGTAAGGATTTC
kanEHind	GATGATGCTAAGCTTCTAAAACAATTCATCCAGTAAAATATA
